# Protein crystal quality oriented disulfide bond engineering

**DOI:** 10.1007/s13238-017-0482-7

**Published:** 2017-10-16

**Authors:** Mengchen Pu, Zhijie Xu, Yao Peng, Yaguang Hou, Dongsheng Liu, Yang Wang, Haiguang Liu, Gaojie Song, Zhi-Jie Liu

**Affiliations:** 1 National Laboratory of Biomacromolecules, Institute of Biophysics, Chinese Academy of Sciences, Beijing, 100101 China; 2 iHuman Institute, ShanghaiTech University, Shanghai, 201210 China; 3 Institute of Biochemistry and Cell Biology, Shanghai Institute of Biological Sciences, Chinese Academy of Sciences, Shanghai, 200031 China; 4 Insititute of Molecular and Clinical Medicine, Kunming Medical University, Kunming, 650500 China; 5 Complex Systems Division, Beijing Computational Science Research Center, Beijing, 100193 China; 6 School of Life Science and Technology, ShanghaiTech University, Shanghai, 201210 China; 7 University of Chinese Academy of Sciences, Beijing, 100049 China

**Dear Editor,**

A disulfide bond that formed between the thiol groups of two spatially close cysteine residues is essential for protein folding, stability, and function (Creighton et al., 1995) (Fass, 2012). Driven by conformational entropy, native disulfide bonds stabilize the conformation of protein molecules (Dill, 1990), while removal of native disulfides usually causes reduced stability of the target protein (Liu and Cowburn, 2016). Previous studies showed that proper introduction of disulfide bonds could stabilize the flexible region of target proteins and reduce the conformational entropy by locking the protein into single desired conformation (Matsumura et al., 1989; Craig and Dombkowski, 2013). Entropy is one of the essential features for protein crystallization (Shaw et al., 2007). Properly engineered disulfide bonds have been shown to decrease protein’s entropy, thus frequently used as strategy for high-resolution structure determination.

G protein-coupled receptors (GPCRs) are a family of membrane proteins that include seven membrane-spanning α-helices (7TM) connected by three loops on each side. Most GPCR crystals were obtained by replacing the N-termini or the 2nd or 3rd intracellular loops with fusion proteins. Introducing a disulfide bond into fusion protein T4 lysozyme (T4L) had not only stabilized the fusion partner itself but also improved the crystal quality of the GPCR-T4L fusion protein (Thorsen et al., 2014). Moreover, disulfide bond has also been applied directly in the extracellular portion of lysophosphatidic acid receptor 1 (LPA1) to solve its high-resolution structure (Chrencik et al., 2015).

To precisely predict sites for disulfide bonds, efforts have been made to analyze the key features and several computational methods have been developed (Ceroni et al., 2006; Tsai et al., 2007). Several software predicted native disulfide bonds with high accuracy (~85%), but predictions of engineered disulfide bonds were not experimentally validated (Ferre and Clote, 2005). Here we developed a comprehensive disulfide bond prediction algorithm that not just increased the success rate of predictions but also improved the quality of crystallized target proteins. New parameters were incorporated in the algorithm, including chemical environment of predicted sites, overall stabilities and conformational entropy changes, the geometric deviations with pre-existing native disulfide bonds in solved high-resolution protein structures. All those parameters were combined into a weighted scoring algorithm where machine learning and data mining of the structures deposited in Protein Data Bank (PDB) were used to train and optimize the weighting scheme. We applied our method on two proteins which were previously determined to high resolution and frequently used as fusion partners for GPCR crystallization, cytochrome b_562_ (BRIL) and Flavodoxin, and verified our prediction by solving the crystal structures of the wild type (WT) proteins and mutants. Furthermore, our algorithm was applied to a previously unsolved GPCR and we successfully solved its high-resolution structure.

We analyzed the features of native disulfide bonds from experimental data set and incorporated the output into our algorithm (Supplementary). These features include: i) the distances between each pair of C, O, N, C_α_, C_β_, S_γ_ atoms and the dihedral angles between each plane of C/C_α_/C_β_, C_α_/C_β_/N, and C/C_α_/N (Fig. S1), ii) the five χ angles (χ^1^, χ^2^, χ^3^, χ^2′^, and χ^1′^) (Fig. S2), iii) the local environment preference of disulfide bonds (Fig. S3). A schematic of the approach is briefly shown in Fig. 1A. For any two given residues, three functions were required for evaluating the possibility of forming disulfide bond between them: 1) *P*_Geom_, the geometrical probability; 2) *P*_RMSD_, the RMSD between predicted disulfide-linked cysteines and the geometrically closest naturally occurring ones in solved structures; 3) *P*_ΔS_, the conformational entropy change induced by the engineered disulfide bond. The details of the calculation are described in Supplementary. Using the *P*_RMSD_, *P*_ΔS_, and *P*_Geom_ calculated from known disulfide bonds as variables, the prediction model was trained and optimized by implementing the Support Vector Machine method (SVM) (Lin and Chang 2011).

**Figure 1.**
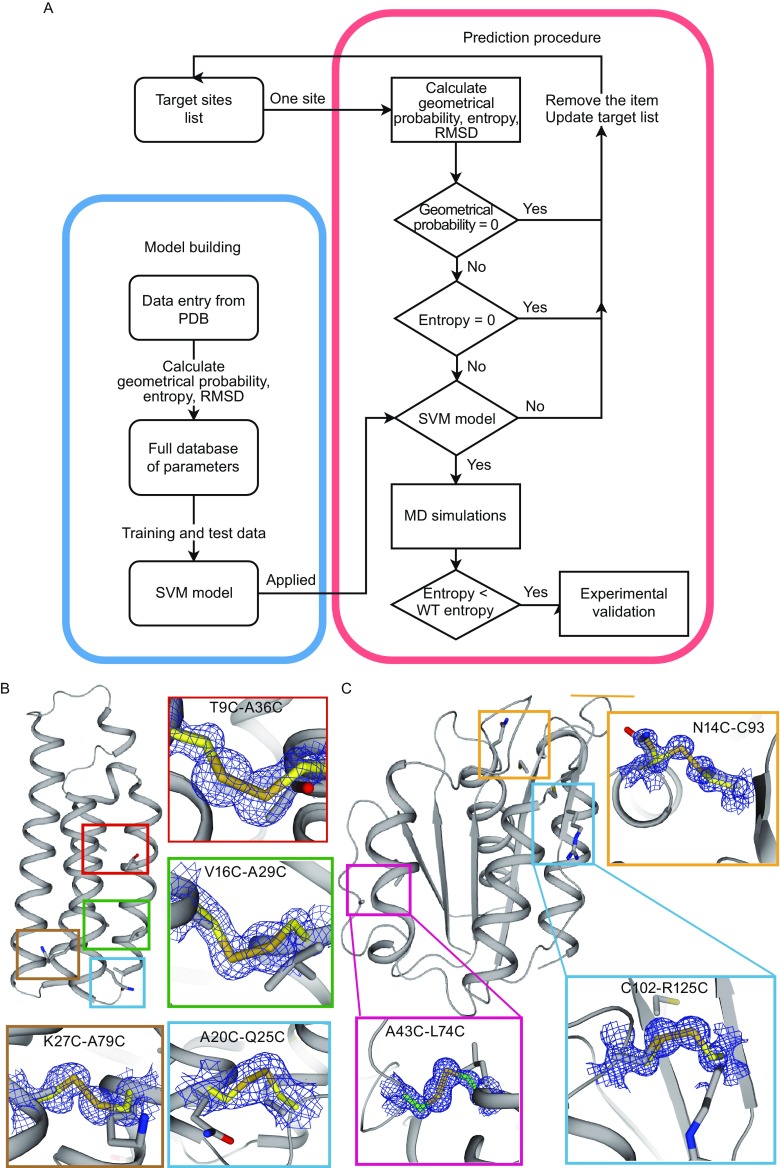
**The prediction algorithm and procedure, and confirmation of disulfide bonds by crystallography**. (A) The models generated from known protein structures are used to predict potential disulfide bond. (B and C) The 2*F*_O_-*F*_C_ electron densities (contoured at 1.5 *σ*) of four pairs of disulfides in BRIL (B) and three pairs of disulfides in Flavodoxin (C). In each box the engineered disulfide bond is aligned with corresponding native residues. Boxes are color coded or linked by solid lines

In order to test our prediction program, two proteins, BRIL and Flavodoxin, were used as examples for disulfide bond engineering. For each target protein, every pair of residues was treated as potential disulfide bond candidate. The *P*_RMSD_, *P*_ΔS_, and *P*_Geom_ were calculated for each pair of candidate residues. Those pairs with either *P*_ΔS_ = 0 or *P*_Geom_ = 0 were removed from candidate list. Then SVM prediction was performed and the final results were sorted by *P*_Geom_. The program generated lists of potential pairs of disulfide bond residues for BRIL and Flavodoxin, separately (Table S1).

A group of top ranked pairs were experimentally verified for both proteins. The mutants were expressed in *E*. *coli* and purified to homogeneity. Eventually, four pairs of BRIL mutants and three pairs of Flavodoxin mutants, together with their WT proteins, were crystallized and structure determined (Tables S4 and S5). The continued disulfide omitted electron densities of the side chains suggested that the disulfide bonds are successfully formed in all crystallized mutants, which are T9C-A36C, A20C-Q25C, V16C-A29C, and K27C-A79C in BRIL and N14C-C93, A43C-L74C, and C102-R125C in Flavodoxin, respectively (Fig. 1B and 1C). In addition, two more disulfide bonds on BRIL were verified by LC/MS, adding our successful rate on BRIL to 6 out of 10 (60%), compared with a web-based prediction tool which yielded 30% successful rate (Table S2).

Compared to WT BRIL, three mutants (T9C-A36C, V16C-A29C, and K27C-A79C) show relatively better or similar diffraction resolution, lower conformational entropy (ΔS) and comparable B-factor (Table 1). These disulfide bonds are located near the middle of helices I and II (T9C-A36C and V16C-A29C) or between helices II and III (K27C-A79C), thus directly strengthened the linkage between these helices (Fig. 1B). In contrast, the other disulfide bond (A20C-Q25C) locates at the edges of helices I and II (Fig. 1B). Insertion of a disulfide bond between these sequentially closed residues may result in distortion of surrounding residues and increase of overall conformational entropy, indicated by its lower resolution (2.2 Å) and much higher B-factor (48.09) (Table 1).

**Table 1:** Data summary of crystallized BRIL, Flavodoxin and their mutants

Mutations	Res (Å)^a^	*T*_m_ (°C)^b^	B (Å^2^)^c^	Δ*S* ^d^	DbD^e^
BRIL_WT	1.56	N/A	22.79		
T9C-A36C	1.3	N/A	26.39	−11.2	Yes
K27-A79C	1.37	N/A	23.04	−24.0	No
V16C-A29C	1.7	N/A	19.71	−11.2	No
A20C-Q25C	2.2	N/A	48.09	−7.8	No
Flavodoxin_WT	1.28	66.99	19.24		
N14C-C93	1.55	69.57	16.15	−31.4	No
A43C-L74C	1.35	61.08	17.95	−25.2	No
C102-R125C	1.5	66.99	18.26	−17.0	Yes

^a^Resolution

^b^Value of thermal stability

^c^B-factor (Å^2^)

^d^Conformational entropy differences compared to WT

^e^Disulfide by Design, a web-based, platform-independent application for prediction of disulfide bond

The WT and disulfide engineered Flavodoxin structures were determined to high-resolution of 1.20–1.55 Å with B-factors in the range of 16.15–19.24 Å^2^ (Table 1). The melting temperature (*T*_m_) for each mutant was measured using the thermo shift assay. Among the three crystallized mutants, N14C-C93 has a significantly improved *T*_m_ value that is three degrees higher than that of WT, and the crystal structure was obtained at 1.55 Å resolution. The mutant C102-R125C, diffracted to 1.5 Å, has a similar *T*_m_ value as the WT protein. A43 is located in a highly dynamic loop region and the disulfide bond A43C-L74C (Fig. 1C) stabilized the loop region (the local B-factor decreased). However, the measured *T*_m_ value decreases by five degrees compared to that of WT, indicating that although local structure can be stabilized by mutations, the overall conformation may be compromised because of the intrinsic connections between different regions within the protein.

To investigate the dynamics of proteins in solvated environments, we carried out all-atom MD simulations and measured features that are relevant to the stability of proteins. For each model (including the WT and mutants), the heavy-atom (carbon, oxygen, nitrogen, and sulfur atoms) root-mean-square deviation (RMSD) with respect to the crystal structure were found to be within 2.0 Å in most cases, suggesting high stability of the structures (Fig. S4A and S4B). The B-factors calculated from simulation trajectories were compared with crystal structure B-factors. For BRIL proteins, the simulation B-factors are consistent with the experimental values (Fig. S4C). For Flavodoxin, the reduced B-factors for R125C mutant indicated that the disulfide bond further stabilizes the structure (Fig. S4D). For BRIL proteins, the distance between the N- and C-termini exhibited small fluctuations expect for K27C-A79C (Fig. S4E). For Flavodoxin, smaller fluctuations of the terminal distances suggest that A43C-74C is more stable among four proteins (Fig. S4F). The conformational entropy was computed using quasi-harmonic approximation (Numata et al., 2007) (Fig. S4G and S4H). The BRIL proteins with disulfide bonds T9C-A36C and K27C-A79C have lower entropy, consistent with better diffraction qualities. On the other hand, the V16C-A29C and A20C-Q25C in BRIL, the N14C-C93 and A43C-L74C in Flavodoxin have higher entropy than that of WT counterparts, consistent with their relatively lower diffraction resolutions.

In addition to the two solved proteins, we also applied our program to an unsolved GPCR. The glucagon-like peptide-1 receptor (GLP-1R) is an important drug target for type 2 diabetes and crystallographers have long been frustrated and failed to solve the structure. We built a model of GLP-1R based on its homolog protein glucagon receptor (GCGR; PDB ID: 4L6R) and predicted disulfide bonds using our algorithm (Table S3). Among the 20 predicted pairs, 6 pairs that cover the potential thermal-dynamic regions are selected for experimental verification. Two pairs of mutants, including the I317C-G361C that remarkably stabilized the receptor (Fig. S5A), were included in the final crystallization construct. The solved structure proved formation of disulfide bond between I317C and G361C (with lower prediction score), whereas the other pair (S193C-M233C) with higher prediction score does not (Fig. S5B). The apparent discrepancy is probably a consequence of the difference between the homolog model from GCGR and the crystal structure of GLP-1R (RMSD 1.6 Å for all Cα), especially the thermal-dynamic region (RMSD 6.1 Å for residue range 193–229 and 274–373).

Our disulfide bond prediction method has shown potential on predicting the disulfide bonds that lead to more stable protein with lower conformational entropy. The theoretical and experimental results both indicated that with relatively lower values of conformational entropy, proteins would be more stable and readily crystallized to higher diffraction resolution (Table 1). Based on our algorithm, the best-engineered constructs of BRIL and Flavodoxin show lower entropy and higher diffraction quality than the wild type proteins, and the predicted disulfide bond on GLP-1R facilitated crystallization of the GLP-1R transmembrane domain. Furthermore, our results indicate that the conformational entropy and protein stability are sensitive to the location of the engineered disulfide bonds. The all-atom molecular dynamics (MD) simulations provide complementary information on thermostability and entropy of the molecules.

In summary, we developed a novel algorithm for prediction of disulfide bonds aiming at high diffraction quality crystals. This algorithm can be utilized as assistive tool for structural determination by X-ray crystallography or single-particle cryo-electron microscopy (Cryo-EM), even for protein design for specific functional states.

## FOOTNOTES

This work was supported by the National Nature Science Foundation of China grant 31330019 (Z.-J.L), 11575021 (H.L.), U1530401 (H.L.), U1430237 (H.L.) and 31500593 (G.S.), the Ministry of Science and Technology of China grants 2014CB910400 (Z.-J.L) and 2015CB910104 (Z.-J.L). This research work is supported by a Tianhe-2JK computing time award at the Beijing Computational Research Center (CSRC). We thank the help on data collection provided from scientists of beamline BL17U1, SSRF. We thank Dr. Jack Skinner for critical reading of the manuscript.

All authors declare that they have no conflict of interest. This article does not contain any studies with human or animal subjects performed by any of the authors.

## Electronic supplementary material

Below is the link to the electronic supplementary material.

Supplementary material 1 (PDF 5907 kb)
