# Supplementary material for: Protein crystal quality oriented disulfide bond engineering
Source: Protein Cell. 2017 Oct 16;9(7):659–63. doi: 10.1007/s13238-017-0482-7 (PMC6019659; doi:10.1007/s13238-017-0482-7)
Supplement: Supplementary file 1 — Supplementary material 1 (PDF 5907 kb) [file 13238_2017_482_MOESM1_ESM.pdf]

Supplementary Figure 1 | The geometric characterization of disulfide bond. A-F, distribution of distances between two paired atoms from disulfide-linked cysteines: C atoms (A), C_α atoms (B), C_β atoms (C), Nitrogen atoms (D), oxygen atoms (E), sulfur atoms (F). According to the optimal dihedral angle (C_β-S_γ-$S_{\gamma'}$-$C_{\beta'}$) of 90°, the C_β distance distribution is very narrow (3.5~4.0 Å), providing a better constrain for disulfide prediction (1C). The distances of the O atoms (7.0-9.0 Å) are less broadly distributed compared with N distances, which could provide extract restrain for the backbone conformation in prediction algorithm. Due to the lack of characteristic distributions, the distance profiles between paired C_α, C and N are less useful in disulfide bond prediction. G-I, the arc length of dihedral angle between planes of $C/C_\alpha/C_\beta$ (G), $C_\alpha/C_\beta/N$ (H), $C/C_\alpha/N$ (I) from each paired cysteine.

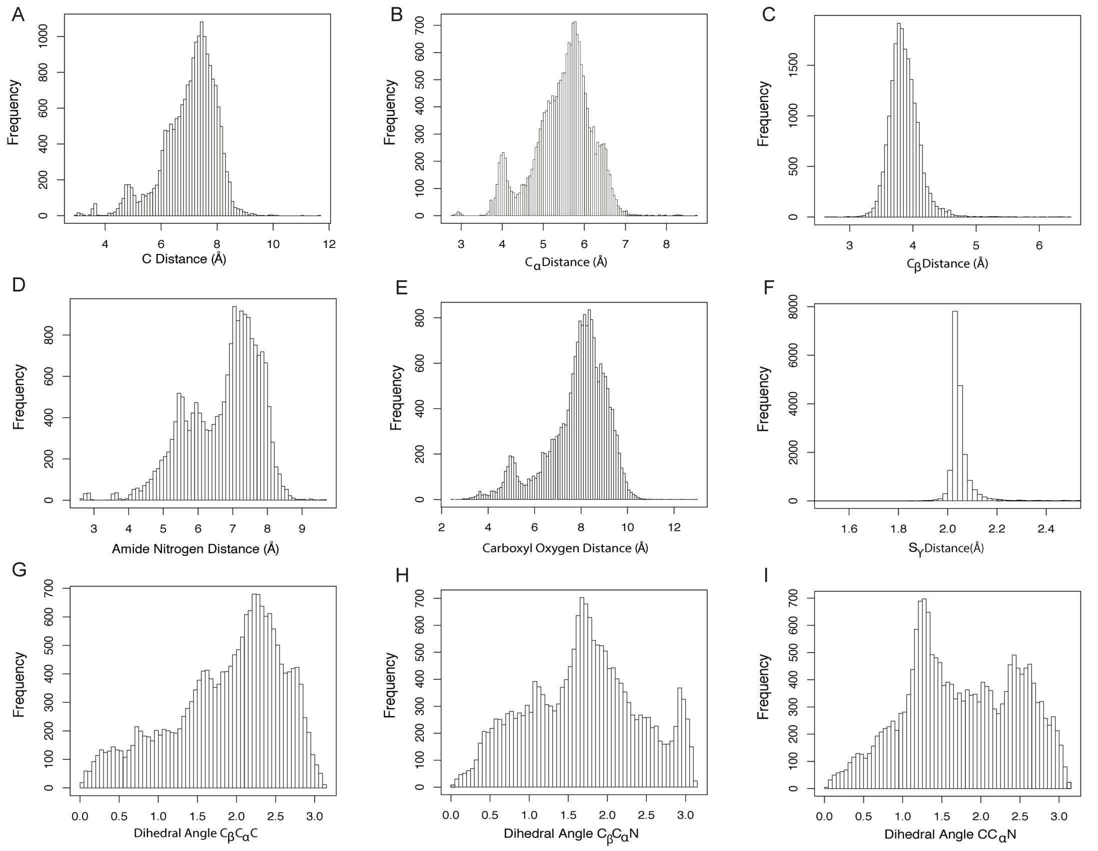

Supplementary Figure 2 | The distribution of χ angles and DSE. A-E, the χ angles (χ1, χ2, χ3, χ2′and χ1′) are rotation angles around the five bonds indicated in the schema Cα-Cβ-Sγ-Sγ′-Cβ′-Cα′, respectively, χ1 (A), χ2 (B), χ3 (C), χ2′ (D), χ1′ (E). F, the DSE distributions.

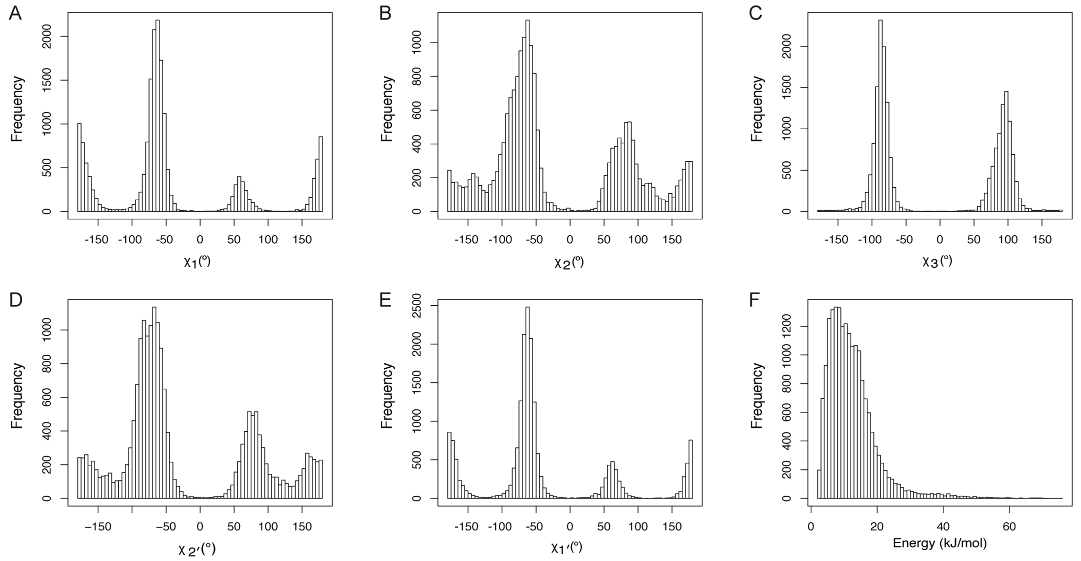

Supplementary Figure 3 | Analysis of environment of disulfide bond. A, statistics of buried and exposed sulfur atoms of disulfide bonds. B, The preference of residues near the disulfide bond. In horizontal axis, amino acids are classified in order: basic amino acid, acidic amino acid, polar amino acid, non-polar amino acid. Residues within 5 Å distance from disulfide sulfurs were calculated. The ratio of exposed to buried is 63/37 (3A). The disulfide linked cysteines are mostly surrounded by nonpolar residues, such as leucine, valine, proline, phenylalanine, and tyrosine (3B). These residues accommodate or cover the disulfide bonds by creating a hydrophobic environment.

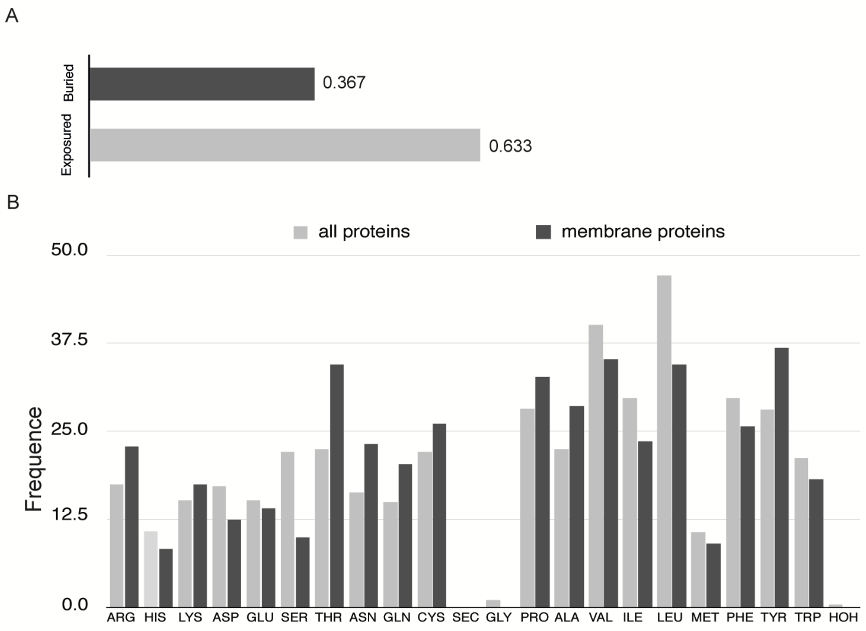

Supplementary Figure 4 | Stability of the proteins from simulations. A, B, Average deviations from crystals structures compared to model resolution for BRIL proteins (A) and Flavodoxin proteins (B). C, D, The average B-factors from simulations and crystal structures for BRIL proteins (C) and Flavodoxin proteins (D). E, F, The distances between two termini of BRIL proteins (E) and Flavodoxin proteins (F). The red columes are the average values computed from simulation trajectories (standard deviation is indicated with error bar), and the yellow columes shows the value measured from crystal structures. G, H，The structural entropy from simulation trajectories of the Bril proteins (G) and Flavodoxin proteins (H) The red columes are structrual entropy computed by Quasi-Harmonic approximation.

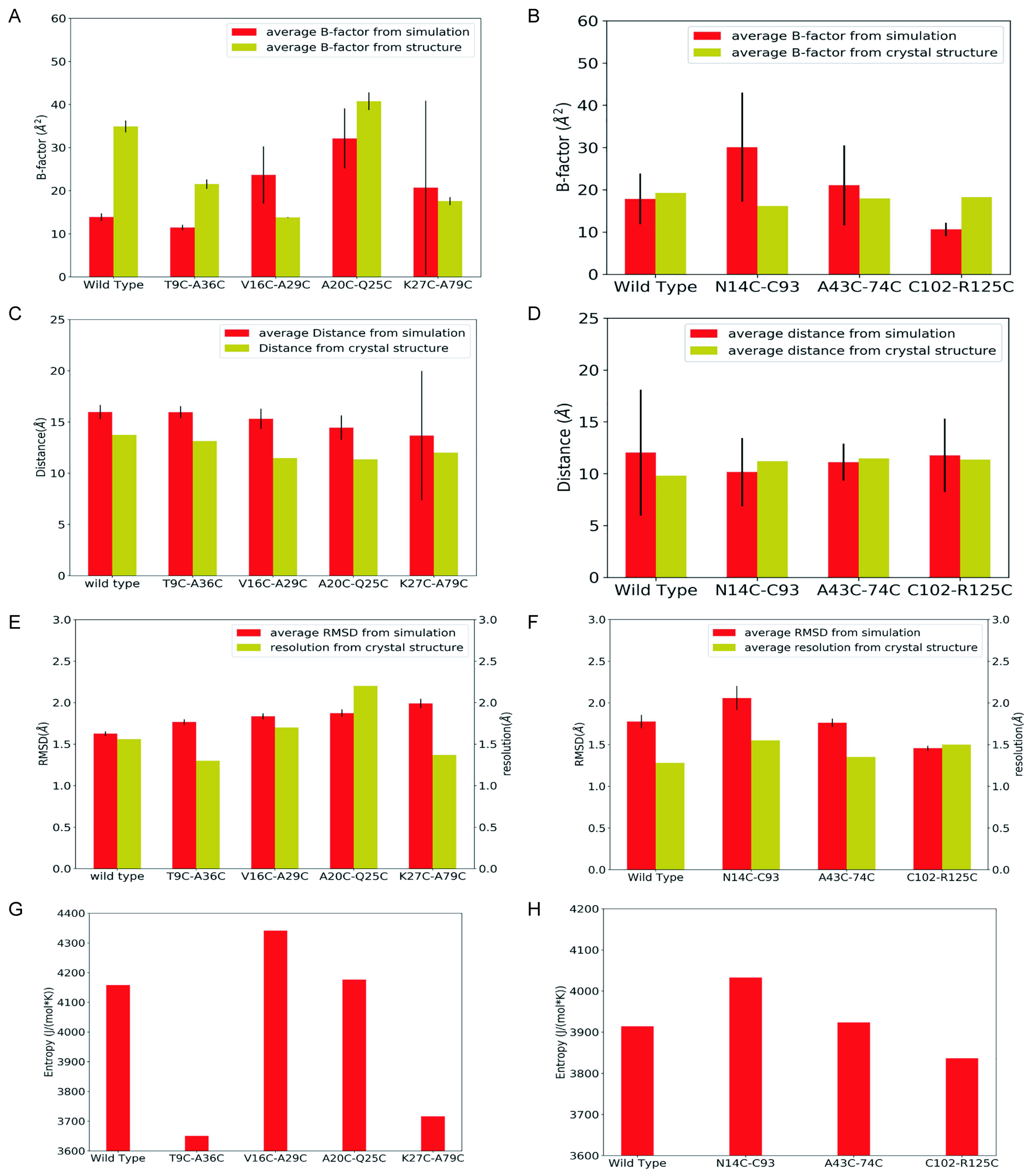

Supplementary Figure 5 | Prediction of disulfide bonds on unknown structure. A, Thermo-SEC characterization of the disulfide I317C-G361C construct. The purified proteins (apo) of I317C-G361C and the control (no mutation) were both kept at 37°C for 5 min and then loaded to the size exclusion chromatography. B, Crystal structure of GLP-1R and the electron densities around the engineered disulfide bonds.

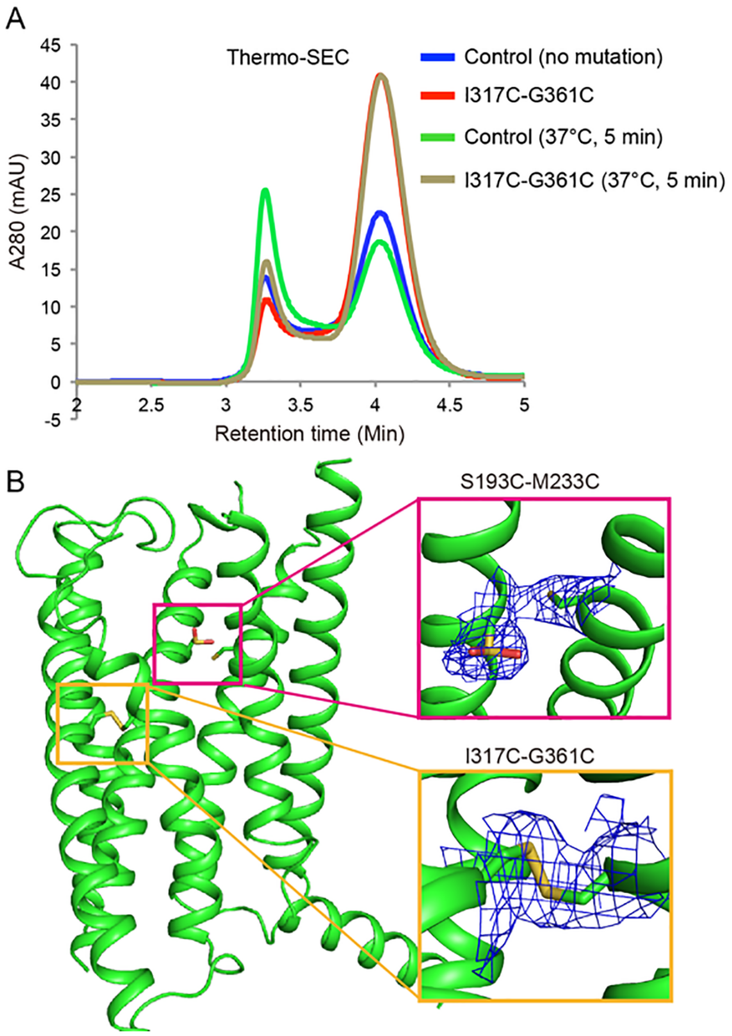

Supplementary table 1| Prediction of disulfide bonds on BRIL and Flavodoxin.

	Mutant site (mutate to cysteine)	Probability (%)
BRIL-WT	—	
Q41C-F65C	41 GLN 65 PHE	27.04
A75C-A90C	75 ALA 90 ALA	26.17
A20C-Q25C	20 ALA 25 GLN	6.90
L78C-A87C	78 LEU 87 ALA	6.01
T9C-A36C	9 THR 36 ALA	0.93
V16C-A29C	16 VAL 29 ALA	0.62
K51C-S55C	51 LYS 55 SER	0.59
A79C-A87C	79 ALA 87 ALA	0.58
K27C-A79C	27 LYS 79 ALA	0.13
S52C-S55C	52 SER 55 SER	0.10
Flavodoxin-WT	—	
R125C-102C	125 ARG 102 CYS	24.00
F50C-L5C	50 PHE 5 LEU	5.40
A104C-T59C	104 ALA 59 THR	5.40
F101C-S96C	101 PHE 96 SER	4.50
N14C-93C	14 ASN 93 CYS	1.40
A43C-L74C	43 ALA 74 LEU	1.20
A104C-57C	104 ALA 57 CYS	1.20
L67C-A104C	67 LEU 104 ALA	1.10

Supplementary Table 2| The results of BRIL and its mutants based on LC/MS.

Label	Molecular weight (MD)[a]	Detected MD [b]	Detected MD （with DTDP） [c]	Formed disulfide bond	DbD[d]
BRIL	11855.29	11855.78	11855.72	-	
Q41C-F65C	11786.27	11784.69	11784.37	YES	YES
A75C-A90C	11919.43	11919.36	11917.49,12137.75	NO	YES
T9C-A36C	11889.40	11887.64	11887.53	YES	YES
V16C-A29C	11891.37	11889.55	11889.52	YES	NO
L78C-A87C	11877.34	11875.44	11875.40	YES	YES
A20C-Q25C	11862.37	11860.50	11860.45	YES	NO
K51C-S55C	11846.33	11845.75	11844.47,12064.60	NO	YES
A79C-A87C	11919.43	11919.49	11917.56,12137.81	NO	YES
K27C-A79C	11862.33	11861.99	11860.48	YES	NO
S52C-S55C	11887.43	11887.76	11886.50,12105.84	NO	YES

Molecular weight of DTDP is 220.3139.

a: The molecular weight of BRIL and mutants with an extra serine in its amino terminal.

b: MD after mutated to cysteine and supposed that mutants didn't form disulfide bond

c: The detected MD by MS experiment.

d: Disulfide by Design, a web-based, platform-independent application for prediction of disulfide bond

Supplementary table 3| Prediction of disulfide bonds on unsolved protein GLP-1R

Protein	Mutant site (mutate to cysteine)		Probability (%)
GLP-1R			
S193C-M233C	193 SER	233 MET	78.40
L183C-W243C	183 LEU	243 TRP	47.10
A162C-403C	162 ALA	403 CYS	46.00
S352C-L401C	352 SER	401 LEU	24.60
S186C-A239C	186 SER	239 ALA	18.10
Y148C-S392C	148 TYR	392 SER	5.60
193C-L232C	193 CYS	232 LEU	4.60
F156C-A191C	156 PHE	191 ALA	3.00
C226-C296#	226 CYS	296 CYS	2.40
A162C-A399C	162 ALA	399 ALA	2.30
L218C-L224C	218 LEU	224 LEU	1.40
A158C-A399C	158 ALA	399 ALA	0.90
I317C-G361C*	317 ILE	361 GLY	0.80
V246C-Y269C	246 VAL	269 TYR	0.70
I147C-S389C	147 ILE	389 SER	0.70
L218C-S223C	218 LEU	223 SER	0.60
Y152C-A191C	152 TYR	191 ALA	0.50
M340C-D344C	340 MET	344 ASP	0.50
S155C-L396C	155 SER	396 LEU	0.50
L144C-F385C	144 LEU	385 PHE	0.40

endogenous disulfide bond

*engineered disulfide bond

Supplementary table 4 | Data collection and refinement statistics (BRIL)

	BRIL (PDB code)	T9C-A36C (PDB code)	V16C-A29C (PDB code)	A20C-Q25C (PDB code)	K27C-A79C (PDB code)
**Data collection**					
Space group	$C222_1$	$C222_1$	$C222_1$	$C12_1$	$C222_1$
Cell dimensions					
a, *b*, *c* (Å)	41.85, 51.16, 89.95	41.76, 50.84, 89.69	40.33, 50.13, 94.24	71.66, 120.87, 95.25	41.77, 51.29, 89.89
a, *β*, *γ* (°)	90.00, 90.00, 90.00	90.00, 90.00, 90.00	90.00, 90.00, 90.00	90.00, 90.04, 90.00	90.00, 90.00, 90.00
Wavelength (Å)	0.9793	0.9793	0.9793	0.9793	0.9793
Resolution (Å)[a]	44.97-1.56 (1.62-1.56)	30.37-1.30 (1.33-1.30)	31.42-1.70 (1.76-1.70)	37.70-2.20 (2.28-2.20)	32.39-1.37 (1.42-1.37)
$R_{merge,}$ (%)	5.8 (21.7)	7.2(43.1)	6.1(10.2)	7.4 (38.7)	7.2 (54.6)
Mean $I/\sigma(I)$	39.06(8.89)	61.49(5.26)	55.71(16.88)	12.35(2.55)	45.75(2.10)
Completeness (%)	99.9(100)	77.1(60.0)	95.4(90.7)	99.0(91.0)	99.5(95.4)
Redundancy	6.9(7.0)	11.6(6.6)	4.4(2.2)	3.7(2.7)	12.8(5.9)
**Refinement**					
Resolution (Å)	44.97-1.56	30.37-1.30	31.42-1.70	37.70-2.20	32.39-1.37
R_{work} (%)/ R_{free}(%)	19.7/23.8	21.3/23.7	15.5/18.8	22.1/26.7	18.1/21.2
Average B factors (Å^2)	22.79	26.39	19.71	48.09	23.04
Protein	21.59	25.48	16.20	48.46	21.48
R.m.s. deviations					
Bond lengths (Å)	0.002	0.006	0.008	0.008	0.008
Bond angles (°)	0.46	0.86	0.71	0.92	0.86
Ramachandran Plot Statistics (%)					
Favored regions	99.04	99.05	100.00	98.00	99.09
Allowed regions	0.96	0.95	0.00	1.70	0.91
Outliners (%)	0.00	0.00	0.00	0.30	0.00

[a] Values in parentheses are for highest-resolution shell.

Supplementary table 5 | Data collection and refinement statistics (Flavodoxin)

	Flavodoxin (PDB code)	N14C-C93 (PDB code)	A43C-L74C (PDB code)	C102-R125C (PDB code)
**Data collection**				
Space group	$P12_11$	$P12_11$	$P12_11$	$P12_11$
Cell dimensions				
a, *b*, *c* (Å)	32.23, 56.26, 41.06	32.61, 56.32, 41.42	32.45, 56.13, 40.95	32.30, 56.07, 41.33
a, β, γ (°)	90.00, 100.84, 90.00	90.00, 101.63, 90.00	90.00, 101.03, 90.00	90.00, 100.56, 90.00
Wavelength (Å)	0.9793	0.9793	0.9793	0.9793
Resolution (Å)[a]	27.59-1.28 (1.32-1.28)	28.16-1.55 (1.58-1.55)	32.68-1.35 (1.40-1.35)	40.63-1.50 (1.55-1.50)
$R_{merge,}$ (%)	8.9(51.7)	19.3(64.9)	9.8(44.0)	11.4(39.7)
Mean $I/\sigma(I)$	32.21(2.72)	21.67(5.77)	26.21(2.73)	15.76(3.33)
Completeness (%)	94.5(90.3)	98.7(98.6)	98.0(96.8)	98.4(99.0)
Redundancy	4.4(3.1)	5.5(4.4)	4.3(2.1)	3.6(3.5)
**Refinement**				
Resolution (Å)	27.59-1.28	28.16-1.55	32.68-1.35	40.63-1.50
R_{work}(%) / R_{free}(%)	16.6/17.7	15.3/18.0	16.7/18.2	17.3/19.3
Average B factors (Å^2)	19.24	16.15	17.95	18.26
Protein	16.39	13.85	15.30	15.40
Ligand	20.96	21.69	17.56	15.86
R.m.s. deviations				
Bond lengths (Å)	0.010	0.016	0.006	0.007
Bond angles (°)	1.12	1.59	0.79	0.90
Ramachandran Plot Statistics (%)				
Favored regions	99.33	99.34	98.70	99.33
Allowed regions	0.67	0.66	1.30	0.67
Outliners (%)	0.00	0.00	0.00	0.00

[a] Values in parentheses are for highest-resolution shell.

MATERIALS AND METHODS

Data sets

We chose 4,722 non-redundant protein structures from PDB with sequence identity <90% that contained at least one disulfide bond. Those structures were obtained using X-ray crystallography and the resolution of those experimental diffraction data is better than 2.5 Å. We then extracted the list of those native disulfide bonds from PDB header files of these non-redundant protein structures. Finally, 18,696 native disulfide bonds were collected from PDB, in which 241 disulfide bonds were from membrane proteins. The data were divided into training (90%) and validation (10%) samples.

Performance measure of the algorithm

The performance of connection prediction was evaluated by inputting the test set into our model and introducing artificial disulfides into BRIL and Flavodoxin. We also took into account the DSE (Yi and Khosla, 2016), and the probability of the neighbor preference to evaluate the output results of our models. The neighbor residue preference and the solvent accessibilities were calculated using Visual Molecular Dynamics (VMD) (Humphrey et al., 1996). To calculate the value of DSE, the form of empirical formula is (Bryan Schmidt, 2006; Schmidt et al., 2006):

$$DSE(kJ/mol) = 8.37\times(1+\cos 3(chi1)) + 8.37\times(1+\cos 3(chi5)) \\ + 4.18\times(1+\cos 3(chi2)) + 4.18\times(1+\cos 3(chi4)) \\ + 14.64\times(1+\cos 2(chi3)) + 2.51\times(1+\cos 3(chi3))$$

The neighbor preference was calculated by

$$Prob_{(enviro)} = \frac{P_1 + P_2 + \cdots + P_N}{N}$$

where N is the order of neighbor residue; P is the probability of occurrence for possible amino acid characterized by statistics analysis; $Prob_{(enviro)}$ is the

probability of the neighbor preference of potential mutant sites.

Prediction algorithm

The P_{Geom} is defined as the following:

$$P_{Geom} = P_{\mathrm{C\beta}} \times P_{\mathrm{O}} \times P_{C/C\alpha/C\beta} \times P_{C\alpha/C\beta/N} \times P_{C/C\alpha/N}$$

where the $P_{\mathrm{C\beta}}$ and P_O are distance distribution probabilities between paired C_β and carbonyl O of disulfide bonding cysteines, respectively, while $P_{\mathrm{C/C\alpha/C\beta}}$, $P_{\mathrm{C/C\alpha/C\beta}}$ and $P_{\mathrm{C\alpha/C\beta/N}}$ represent the dihedral angle distribution probabilities between paired corresponding planes. $P_{\Delta\mathrm{S}}$ is defined as the following:

$$P_{\Delta S} = -2.1 - \frac{3}{2} R ln(n)$$

where n is the number of residues closed by loop resulted by the new disulfide bond and R is the universal gas constant (Pace et al., 1988).

The clustering of geometrical difference of disulfide bonds from the PDB was detected by MMTSB (Multiscale Modeling Tools for Structural Biology) and assessed by RMSD (Feig et al., 2004). The formula for calculating P_{RMSD} is：

$$P_{RMSD} = \sqrt{\frac{\sum_{i=1}^{n} \left(X_{obs,i} - X_{model,i}\right)^2}{n}}$$

where $X_{obs,i}$ is the object value, $X_{model,i}$ is the value from the statistics analysis and n represent the number of structure of disulfide bonds from our data set. By using hierarchical clustering algorithm based on RMSD, six clusters from the dataset were generated. The RMSD between those six cluster centers and the target positions are calculated.

Molecular Dynamics Simulations

To investigate the changes of the mutated BRIL and Flavodoxin structures under room temperature 303K, we performed all atom molecular dynamics simulations for each system. The crystal structures were used as the starting conformation, and the

simulation systems were constructed using Charmm-GUI Webserver (Jo et al., 2008; Lee et al., 2016). Constant pressure and temperature (NPT) MD simulations were performed with integration time step of 2 femtoseconds. Charmm36 force field (Mallajosyula et al., 2012) was used for the modeling of systems, and simulation trajectories were generated by using Gromacs 5.1.2 (Berendsen et al., 1995; Lindahl et al., 2001). The rectangular water box was added to ensure that the distance from protein to edge of box is 20 Å. We used TIP3P as the water model to solvate protein systems. Sodium chloride ions were added neutralize the systems and excess ions were used to obtain ion concentration of 150 mM NaCl. The temperature is controlled by Nosé-Hoover thermostat at 303K (Nosé, 1984; Hoover, 1985). The disulfide bonds for mutants were connected manually to generate the appropriate models. The simulation trajectories are equivalent to 200 nano seconds for each system.

Plasmid construction

The full-length BRIL and Flavodoxin genes were amplified from pfastbac plasmid (synthetized by GENEWIZ). The resultant gene fragments were ligated into pMCSG7 encoding His-TEV-BRIL and His-TEV-Flavodoxin. Mutations were designed based on this plasmid pMCSG7-BRIL and pMCSG7-Flavodoxin by using a QuikChange™ site-directed mutagenesis kit following the manufacturer's instructions (Stratagene, La Jolla, CA, USA). All the plasmids were confirmed by sequencing. The construct, expression, purification and crystallization of GLP-1R were described elsewhere (Song et al., 2017).

Protein Purification

Escherichia coli strain BL21-DE3 containing plasmid of protein with His-tag and mutations were grown while shaking (250 rpm) at 37°C in 1L of Luria–Bertani medium supplemented with ampicillin (100 µg/mL) until the optical density at 600

nm reached 1.2–1.5, at which time 1 mM isopropy-β-D-thiogalactoside was added. The culture was grown overnight at 16°C and harvested by centrifugation for 30 min at 6000×*g*. The cell pellet was resuspended in 40 mL of 50 mM Tris-HCl and 150 mM NaCl buffer at pH 8.0. The cells were then lysed by sonication (Noise Isolation Chamber, SCIENTZ), and the lysate was cleared by centrifugation at 20,000 rpm for 30 min. Cell lysate was applied onto a Ni column equilibrated with 50 mM Tris-HCl and 150 mM NaCl buffer at pH 8.0, and the Ni column was washed with 3 column volume (CV) washing buffer A containing 50 mM Tris-HCl, 150 mM NaCl and 30 mM imidazole, and 3 CV washing buffer B containing 50 mM Tris-HCl, 150 mM NaCl and 50 mM imidazole. The protein was then eluted with elution buffer containing 50 mM Tris-HCl, 150 mM NaCl and 300 mM imidazole. The eluted solution was desalted and concentrated by ultrafiltration in an Amicon cell with a 3K MWCO membrane, YM3 (Amicon) in 50 mM Tris-HCl and 150 mM NaCl at pH 8.0. Then the TEV protease was added into the protein solution with a molar rate of 1:50, and placed at 4°C overnight.

After detected by protein gel electrophoresis, the protein and TEV protease mixture was added into a new Ni column and the protein was collected in the flow through, then the protein was concentrated to less than 1 ml before loaded to the gel filtration column Superdex 75 10/300GL column (GE Healthcare). The purified homogenous BRIL and Flavodoxin proteins were collected and concentrated to 15-25 mg/ml in buffer 50 mM Tris-HCl (pH 8.0) and 20 mM NaCl.

Crystallization and data collection

The WT BRIL and its mutants were crystallized by the hanging drop diffusion technique. The protein, at a concentration of 15-25 mg/ml, was stored in 50 mM Tris-HCl (pH 8.0) and 20 mM NaCl. The well solution contains 3.2 M ammonium sulfate,

0.1 M bicine (pH 9.0) N-octanoylsucrose (Reagent 17 of the Detergent Screen, Hampton Research) was added to the crystallization drops to a final concentration of 2.44 mM (Chu et al., 2002). The final volume of crystallization drops was 4 μl, containing protein solution, well solution, and additive solution at a ratio of 5:4:1 (by vol.). The WT Flavodoxin and its mutants were also crystallized by the hanging drop diffusion technique. Crystals of Flavodoxin were grown in a buffer containing 3.1 M or 3.2 M $(NH_4)_2SO_4$ and 0.1 M Tris-HCl pH 7.5 or pH 7.0 (Ross A. Reynolds, 2001). High diffraction crystals of Flavodoxin appeared after 3-4 weeks with concentrations between 15 and 20 mg/ml. Crystals were frozen in liquid nitrogen prior to diffraction testing and data collection.

Data processing and structure determination

Native diffraction data were collected at a wavelength of 0.979 Å at beamline BL17U1 at SSRF. The dataset was indexed, integrated, and scaled using the HKL2000 software package (Otwinowski and Minor, 1997). The structure was solved by molecular replacement method (McCoy et al., 2007) using wild-type BRIL (PDB:1M6T) and Flavodoxin (PDB: 1J8Q) as search models respectively. Refinement was carried out using PHENIX Refine (Adams et al., 2010). The refinement parameters were summaried in Supplementary Table 1 and 2.

Mass Spectrum experiment

The protein samples were diluted with PBS to adjust to final disulfide concentration of 40 μM in 1 ml volume. After addition of 50 μl 4 mM DTDP reagent, the sample was immediately mixed and the mix sample reacted in room temperature for 2 h. Then the sample was injected into 6320 TOF LC/MS system (Agilent Technologies, USA). The data were analyzed by using Agilent MassHunter Qualitative analysis.

Adams, P.D., Afonine, P.V., Bunkoczi, G., Chen, V.B., Davis, I.W., Echols, N., Headd, J.J., Hung, L.W., Kapral, G.J., Grosse-Kunstleve, R.W., *et al.* (2010). PHENIX: a comprehensive Python-based system for macromolecular structure solution. Acta Crystallogr D Biol Crystallogr 66, 213-221.
Berendsen, H.J.C., van der Spoel, D., and van Drunen, R. (1995). GROMACS: A message-passing parallel molecular dynamics implementation. Comp Phys Comm 91, 43-56.
Bryan Schmidt, L.H., Philip J. Hogg (2006). Allosteric Disulfide Bonds. Biochemistry 45, 5.
Chu, R., Takei, J., Knowlton, J.R., Andrykovitch, M., Pei, W., Kajava, A.V., Steinbach, P.J., Ji, X., and Bai, Y. (2002). Redesign of a four-helix bundle protein by phage display coupled with proteolysis and structural characterization by NMR and X-ray crystallography. J Mol Biol 323, 253-262.
Feig, M., Karanicolas, J., and Brooks, C.L., 3rd (2004). MMTSB Tool Set: enhanced sampling and multiscale modeling methods for applications in structural biology. J Mol Graph Model 22, 377-395.
Hoover, W.G. (1985). Canonical dynamics: Equilibrium phase-space distributions. Phys Rev A Gen Phys 31, 1695-1697.
Humphrey, W., Dalke, A., and Schulten, K. (1996). VMD: visual molecular dynamics. J Mol Graph 14, 33-38, 27-38.
Jo, S., Kim, T., Iyer, V.G., and Im, W. (2008). CHARMM-GUI: a web-based graphical user interface for CHARMM. J Comput Chem 29, 1859-1865.
Lee, J., Cheng, X., Swails, J.M., Yeom, M.S., Eastman, P.K., Lemkul, J.A., Wei, S., Buckner, J., Jeong, J.C., Qi, Y., *et al.* (2016). CHARMM-GUI Input Generator for NAMD, GROMACS, AMBER, OpenMM, and CHARMM/OpenMM Simulations Using the CHARMM36 Additive Force Field. J Chem Theory Comput 12, 405-413.
Lindahl, E., Hess, B., and van der Spoel, D. (2001). GROMACS 3.0: A package for molecular simulation and trajectory analysis. J Mol Mod 7, 306-317.
Mallajosyula, S.S., Guvench, O., Hatcher, E., and Mackerell, A.D., Jr. (2012). CHARMM Additive All-Atom Force Field for Phosphate and Sulfate Linked to Carbohydrates. J Chem Theory Comput 8, 759-776.
McCoy, A.J., Grosse-Kunstleve, R.W., Adams, P.D., Winn, M.D., Storoni, L.C., and Read, R.J. (2007). Phaser crystallographic software. J Appl Crystallogr 40, 658-674.
Nosé, S. (1984). A unified formulation of the constant temperature molecular-dynamics methods. Journal of Chemical Physics 81, 511-519.
Otwinowski, Z., and Minor, W. (1997). [20] Processing of X-ray diffraction data collected in oscillation mode. Methods Enzymol 276, 307-326.
Pace, C.N., Grimsley, G.R., Thomson, J.A., and Barnett, B.J. (1988). Conformational stability and activity of ribonuclease T1 with zero, one, and two intact disulfide bonds. J Biol Chem 263, 11820-11825.
Ross A. Reynolds, a.W.W., Keith D. Watenpaughb (2001). Structures and comparison of the Y98H (2.0 Å) and Y98W (1.5 Å) mutants of flavodoxin (Desulfovibrio vulgaris). Acta Cryst 57, 9.
Schmidt, B., Ho, L., and Hogg, P.J. (2006). Allosteric disulfide bonds. Biochemistry 45, 7429-7433.
Song, G., Yang, D., Wang, Y., de Graaf, C., Zhou, Q., Jiang, S., Liu, K., Cai, X., Dai, A., Lin, G., *et al.* (2017). Human GLP-1 receptor transmembrane domain structure in complex with allosteric modulators. Nature 546, 312-315.
Yi, M.C., and Khosla, C. (2016). Thiol-Disulfide Exchange Reactions in the Mammalian Extracellular Environment. Annu Rev Chem Biomol Eng 7, 197-222.
